# A Unified AI-Driven Multimodal Framework Integrating Visual Sensing and Wearable Sensors for Robust Human Motion Monitoring in Biomedical Applications

**DOI:** 10.3390/s26082314

**Published:** 2026-04-09

**Authors:** Qiang Chen, Xiaoya Wang, Ranran Chen, Surui Hua, Yufei Li, Siyuan Liu, Yan Zhan

**Affiliations:** 1 Department of Physical Education and Military Affairs, China Jiliang University, Hangzhou 310018, China; 2 National School of Development, Peking University, Beijing 100871, China; 3 China Agricultural University, Beijing 100083, China; 4 Artificial Intelligence Research Institute, Tsinghua University, Beijing 100084, China

**Keywords:** temporal synchronization, kinematic-physiological fusion, uncertainty-aware weighting, wearable sensor integration, pose estimation

## Abstract

This study proposes a unified multimodal temporal motion state perception framework for optical imaging-oriented biomedical applications, integrating visual skeleton sequences, inertial measurement unit (IMU) signals, and surface electromyography (EMG) signals. The framework utilizes modality-specific encoders and a cross-modal temporal alignment attention mechanism to explicitly model temporal offsets from heterogeneous sensing streams. A multimodal temporal Transformer backbone is introduced to capture long-range motion dependencies and cross-modal interactions, while an uncertainty-aware fusion module dynamically allocates weights based on modality confidence. Experimental results demonstrate that the proposed approach achieves an accuracy of 94.37%, an F1-score of 93.95%, and a mean average precision of 96.02%, outperforming mainstream baseline models. Robustness evaluations further confirm stable performance under visual occlusion and sensor noise. These results indicate that the framework provides a highly accurate and robust solution for rehabilitation assessment, sports training monitoring, and wearable intelligent interaction systems.

## 1. Introduction

The primary objective of this study is to develop a unified AI-driven multimodal framework that achieves robust human motion monitoring by resolving the inherent challenges of data heterogeneity and temporal misalignment. Human motion state perception represents a critical research direction in intelligent sensing and human–computer interaction, demonstrating extensive application value in motion analysis, rehabilitation training assessment [1,2], and intelligent medical monitoring [3,4,5]. However, the precise modeling of these states through objective, automated approaches requires bridging the gap between high-level semantic recognition and low-level biomechanical signal integration. Therefore, rather than focusing solely on generic perception models, this work directs its efforts toward the architectural synchronization of visual and wearable streams to establish a reliable foundation for smart healthcare systems [6,7].

Traditional human motion analysis methods have evolved from classical state estimation to sophisticated data-driven frameworks [8,9]. Early sensor fusion techniques, such as those based on Kalman filters and their variants, were widely employed to achieve real-time state estimation and noise reduction by fusing linear or near-linear kinematic signals [10]. While Kalman filters provide a mathematically rigorous approach for sensor fusion, they often encounter limitations when handling high-dimensional, non-linear human skeletal sequences and the stochastic nature of electromyography (EMG) signals. Consequently, the field has transitioned toward deep learning paradigms. Vision-based methods primarily perform human detection and pose estimation through video imagery [11,12], utilizing convolutional neural networks (CNNs) and graph convolutional networks (GCNs) to model skeletal topology [13,14,15]. However, these methods remain vulnerable to occlusion and lack observability of internal biomechanical dynamics [16,17,18,19].

To address these limitations, wearable sensor-based methods (IMU/EMG) provide continuous signals with high temporal resolution [20,21]. IMUs capture limb dynamics, while EMG signals reveal neuromuscular control mechanisms [22,23]. While RNNs and TCNs have strengthened temporal modeling [24,25], wearable sensors struggle to represent spatial structural relationships [26,27,28]. Consequently, multimodal motion analysis has emerged as a research hotspot, aiming to fuse visual information with sensor signals for a complementary representation [29,30]. Modern architectures, particularly Transformer models, have demonstrated outstanding performance in capturing cross-modal interactions [31,32,33]. Recent frameworks like Husformer [34] and MTFT [35] have attained significant gains in recognition accuracy [36,37]. A comprehensive assessment of current state-of-the-art (SOTA) methods regarding their fusion strategies and handling of frequency heterogeneity is summarized in Table 1.

Despite these advancements, critical research gaps remain. First, inconsistent sampling rates across modalities make temporal synchronization difficult. Second, visual errors and sensor noise often become coupled and amplified during fusion. Third, existing fusion strategies lack the capability to model modality uncertainty under varying data quality [38,39,40]. Therefore, achieving cross-modal alignment within a unified temporal semantic space has become the core issue [41].

To fill these gaps, this study proposes a unified multimodal temporal Transformer framework. We adopt unified temporal modeling as the core paradigm and design a cross-modal temporal alignment attention mechanism to dynamically match heterogeneous data with different sampling rates and temporal offsets. Furthermore, we introduce an uncertainty-aware fusion strategy that estimates modality reliability and adaptively allocates weights. The main contributions are:

1. We propose a unified multimodal Transformer framework for joint representation learning of vision-based skeletons, IMU signals, and EMG signals, bridging the gap between high-level spatial structure and low-level biomechanical dynamics.
2. We develop a cross-modal temporal alignment attention mechanism utilizing learnable offsets to resolve asynchronous timing discrepancies inherent in heterogeneous data streams with significantly different sampling rates.
3. We introduce an uncertainty-aware fusion strategy that dynamically allocates modality weights based on predictive reliability, ensuring system robustness under conditions of visual occlusion or sensor noise.
4. We establish a rigorous validation benchmark on a 9216-sample dataset, providing empirical evidence that the framework maintains superior stability and synchronization accuracy in complex, real-world motion scenarios.

## 2. Materials and Method

### 2.1. Data Collection

In this study, a multimodal human motion state perception dataset was constructed through a self-established experimental acquisition platform. The data sources comprised three modalities, including visual video, inertial measurement unit signals, and surface electromyography signals, aiming to collaboratively characterize human motion states from the perspectives of spatial structure, kinematic variation, and muscle activation mechanisms, as shown in Table 2. To address the significant discrepancy in sampling frequencies—ranging from 30 Hz for video to 1000 Hz for EMG—a unified hardware synchronization strategy was implemented. A central synchronization controller (master clock) was utilized to issue a simultaneous start-trigger pulse to the camera system, the IMU network, and the EMG acquisition device at the beginning of each session. This hardware-level triggering ensured that all modality streams shared a common global time zero.

The data acquisition campaign was conducted in multiple phases from March 2024 to November 2024. Experimental sites were arranged in both a standard indoor motion laboratory and a rehabilitation training assessment center. Experimental tasks included continuous action recognition, motion phase segmentation, and posture stability evaluation. Visual data were collected using a multi-view RGB camera system at 30 fps. For temporal alignment during multimodal fusion, the maximum temporal synchronization error across all sensors was measured to be less than 1 ms, which is significantly finer than the 33.3 ms interval of the video frames. In the post-processing stage, to reconcile the heterogeneous sampling rates, skeletal keypoints extracted at 30 Hz and IMU data at 100 Hz were upsampled to a unified 1000 Hz temporal grid using cubic spline interpolation, aligned to the high-resolution EMG timestamps.

IMU data were collected through wearable nodes at 100 Hz. Raw signals were recorded with timestamps anchored to the master clock to ensure cross-modal temporal consistency. EMG data were acquired using a multi-channel system at 1000 Hz. To reduce noise, skin surfaces were cleansed, and band-pass filtering was applied. By maintaining a sub-millisecond synchronization accuracy, the framework ensures that rapid muscle activation spikes in the EMG signals are precisely correlated with the corresponding kinematic variations and spatial posture changes, effectively mitigating the alignment artifacts often encountered in high-frequency heterogeneous data fusion.

All participants were healthy adult volunteers, totaling 32 individuals, with a balanced gender ratio and an age distribution ranging from 22 to 35 years. The demographic characteristics of the participants, including sex, height, and weight distribution, are summarized in Table 3.

Each participant completed 3–5 acquisition rounds for all motion sequences, with each round lasting approximately 6–10 min, resulting in more than 85 h of effective recorded data. A three-level aligned annotation strategy integrating video, skeleton, and sensor streams was adopted. Action categories and motion phases were first annotated at the video frame level, followed by temporal mapping to corresponding IMU and EMG signal segments. A unified temporal semantic labeling system was thereby established, providing high-quality supervisory information for subsequent multimodal joint modeling.

### 2.2. Data Preprocessing and Augmentation Strategy

In the multimodal temporal human motion state perception framework, data preprocessing and data augmentation—a strategy used to artificially increase the diversity and volume of the training set by applying various transformations to existing samples—are not merely engineering procedures for performance improvement but also critical theoretical processes that ensure multi-source signals are comparable and fusion-compatible within a unified semantic space. In alignment with the primary objective of this study to establish a robust and high-precision monitoring framework, these steps ensure that discrepancies across modalities in terms of sampling mechanisms, physical meanings, noise distributions, and scale ranges are addressed. Without unified normalization and temporal consistency processing, subsequent cross-modal attention modeling would struggle to learn stable mapping relationships. Therefore, preprocessing is conducted from both visual and sensor modalities, combined with temporal alignment and resampling strategies, to construct unified multimodal temporal representations.

During the video data preprocessing stage, human detection algorithms are first employed to localize target regions. Let the raw video frame be denoted as $I_{t}\;\in \;R^{H\times W\times 3}$, where *t* represents the temporal index and *H* and *W* denote image height and width, respectively. Through the human detection model, a bounding box $B_{t}\;=\;\left(x_{t},\;y_{t},\;w_{t},\;h_{t}\right)$ is obtained, enabling the cropped human-region image $I_{t}^{crop}$ to be extracted. Subsequently, a pose estimation network (specifically, the HRNet-w32 architecture) is applied to extract keypoint coordinates. The skeletal keypoint set of the *t*-th frame is denoted as $S_{t}\;=\;{\left\{\left(x_{t,i},\;y_{t,i}\right)\right\}}_{i=1}^{J}$, where *J* represents the number of joints. To eliminate scale variations caused by inter-subject differences and camera distance changes, keypoint normalization is required. A common approach adopts torso length or keypoint centroid as the reference scale, formulated as

(1) $${\tilde{x}}_{t,i}\;=\;\frac{x_{t,i}\;-\;\mu_{t}^{x}}{\sigma_{t}},\;{\tilde{y}}_{t,i}\;=\;\frac{y_{t,i}\;-\;\mu_{t}^{y}}{\sigma_{t}},$$

where $\mu_{t}^{x}$ and $\mu_{t}^{y}$ denote the centroid coordinates of keypoints in the current frame, and $\sigma_{t}$ represents the scale factor, such as shoulder width or torso length. Through this normalization operation, different video sequences are mapped into a unified spatial scale, reducing spatial distribution discrepancies that may interfere with model training. Each frame skeleton is ultimately represented as ${\tilde{S}}_{t}\;\in \;R^{J\times 2}$, and a temporal skeleton sequence $S\;=\;{\left\{{\tilde{S}}_{t}\right\}}_{t=1}^{T}$ is constructed as the visual modality input.

### 2.3. Proposed Method

#### 2.3.1. Overall Architecture

After temporal alignment and feature preparation of multimodal data, an end-to-end processing pipeline is constructed at the model design level, consisting of modality-specific encoding, cross-modal temporal alignment, unified temporal modeling, uncertainty-aware fusion, and task prediction. Let the aligned visual skeleton sequence be $S\;=\;{\left\{{\tilde{S}}_{t}\right\}}_{t=1}^{T}\;\in \;R^{T\times J\times 2}$, where *J* is the number of joints. Similarly, let the IMU sequence be $I\;=\;{\left\{i_{t}\right\}}_{t=1}^{T}\;\in \;R^{T\times C_{I}}$ and the EMG sequence be $E\;=\;{\left\{e_{t}\right\}}_{t=1}^{T}\;\in \;R^{T\times C_{E}}$, where $C_{I}$ and $C_{E}$ represent the number of inertial and electromyography channels, respectively. These sequences share a unified temporal index *t*.

The three modalities are first processed by dedicated modality-specific encoders to project these heterogeneous features into a shared latent space with a unified embedding dimension *d*. This identical dimensionality *d* ensures that features from different sources can be directly interacted with and compared within the subsequent attention-based modules. The skeleton encoder extracts spatial structural information and local motion patterns from joint coordinate sequences, producing $F_{S}\;\in \;R^{T\times d}$. The IMU encoder refines temporal kinematic characteristics from acceleration and angular velocity sequences, yielding $F_{I}\;\in \;R^{T\times d}$. The EMG encoder captures muscle activation intensity and rhythmic dynamics, generating $F_{E}\;\in \;R^{T\times d}$.

To explicitly address potential fine-grained temporal delays and semantic misalignment across modalities, a cross-modal temporal alignment attention module is introduced. Anchored on visual features at each time step *t*, the most relevant temporal context is adaptively selected from neighboring sensor segments, resulting in softly aligned representations ${\hat{F}}_{I}$ and ${\hat{F}}_{E}$, which are combined with $F_{S}$ to form temporally consistent joint representations. The fused features are then fed into a multimodal temporal Transformer backbone, where long-range intra-modal dependencies and cross-modal interactions are jointly modeled within a shared attention space, producing high-level temporal semantic representations $H\;=\;{\left\{h_{t}\right\}}_{t=1}^{T}$. During the fusion stage, an uncertainty-aware module estimates modality reliability at each time step and dynamically assigns weights to aggregate multimodal features, suppressing noisy modalities while enhancing reliable ones. A robust global motion state representation $Z\;=\;{\left\{z_{t}\right\}}_{t=1}^{T}$ is thereby obtained. Finally, Z is passed to task-specific prediction heads to generate action categories, motion phases, or stability scores, enabling stable and generalizable human motion state perception under complex motion scenarios.

#### 2.3.2. Cross-Modal Temporal Alignment Attention Module

The cross-modal temporal alignment attention module is positioned before the unified temporal modeling backbone, aiming to explicitly mitigate implicit temporal misalignment between visual skeleton sequences and wearable sensor sequences caused by sampling discrepancies, neuromuscular response latency, and device clock drift. Unlike conventional self-attention mechanisms that model temporal dependencies within a single modality, the proposed module adopts cross-modal and cross-temporal neighborhood attention as the fundamental computational unit. The visual modality serves as a temporal anchor, while sensor modalities are softly matched within local temporal windows to establish explicit alignment in a unified semantic space.

**Figure 1:**
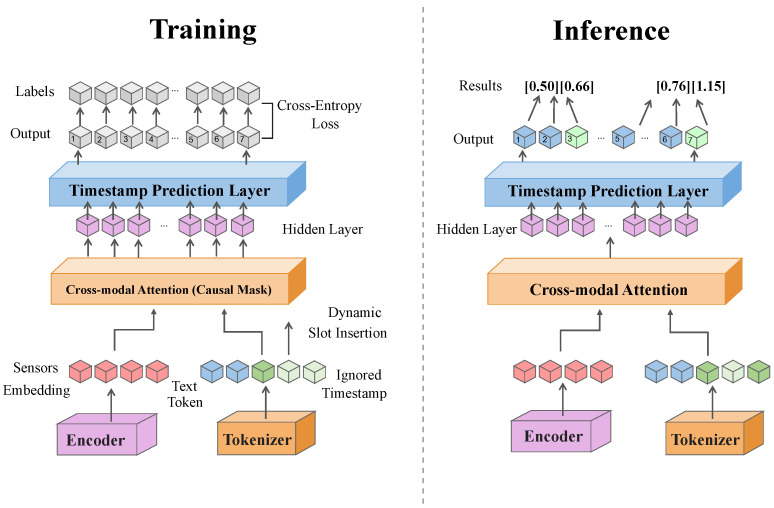
Schematic illustration of the cross-modal temporal alignment attention module, depicting dynamic soft alignment between visual skeleton and sensor sequences within local temporal neighborhoods via cross-modal attention.

As shown in Figure 1, the encoded visual representation is denoted as $F_{S}\;\in \;R^{T\times C}$, and the encoded IMU and EMG representations are denoted as $F_{I},\;F_{E}\;\in \;R^{T\times C}$, where *T* represents temporal length, and *C* denotes channel dimensionality. The module adopts a hierarchical stacked architecture composed of linear projection sublayers, relative temporal position encoding sublayers, and multi-head cross-modal attention sublayers. Through parallel attention heads, temporal offset patterns and cross-modal semantic correlations are learned within different subspaces, enhancing alignment resolution and modeling robustness. For a visual feature at time step *t*, a query vector is generated via linear projection, while sensor features within a local temporal neighborhood centered at *t* are projected to keys and values. The cross-modal temporal alignment attention is computed as follows:

(2) $$Q_{t}\;=\;W_{q}F_{S}^{t},\;K_{k}\;=\;W_{k}F_{M}^{k},\;V_{k}\;=\;W_{v}F_{M}^{k},$$

(3) $$A_{t,k}\;=\;\frac{\exp\left(\frac{Q_{t}K_{k}^{⊤}}{\sqrt{d}}\right)}{\sum_{k^{\prime }\in \Omega_{t}}\exp\left(\frac{Q_{t}K_{k^{\prime }}^{⊤}}{\sqrt{d}}\right)},$$

(4) $${\hat{F}}_{M}^{t}\;=\;\sum_{k\in \Omega_{t}}A_{t,k}V_{k},$$

where M ∈ {I, E} denotes the sensor modality and $\Omega_{t}$ represents the temporal neighborhood set. Compared with standard self-attention,

(5) $$\mathrm{SelfAttn}\left(X\right)\;=\;\mathrm{Softmax}\left(\frac{QK^{⊤}}{\sqrt{d}}\right)V,$$

the proposed mechanism decouples query and key-value sources across modalities and restricts attention computation to a local temporal domain. Consequently, the objective of attention learning shifts from generic sequence dependency modeling to explicit temporal offset estimation. Mathematically, this process can be interpreted as learnable dynamic time warping along the temporal axis, where the attention distribution functions as an implicit probability density over temporal alignment positions. To further enhance temporal compensation capability, a learnable temporal offset parameter is introduced and incorporated via relative positional encoding into the attention calculation:

(6) $$K_{k}^{\prime }\;=\;K_{k+\delta_{M}},\;V_{k}^{\prime }\;=\;V_{k+\delta_{M}},$$

(7) $$A_{t,k}^{align}\;=\;\mathrm{Softmax}\left(\frac{Q_{t}{\left(K_{k}^{\prime }\;+\;R_{t-k}\right)}^{⊤}}{\sqrt{d}}\right),$$

where $\delta_{M}$ denotes the learnable temporal offset and $R_{t-k}$ represents the relative temporal encoding term. According to the expectation alignment property of attention, when the distribution satisfies

(8) $$E\left[k-t\right]\;=\;\delta_{M},$$

the aligned output achieves optimal temporal matching under the mean squared error criterion, thereby minimizing cross-modal semantic misalignment. After stacked alignment layers, temporally consistent features ${\hat{F}}_{I}$ and ${\hat{F}}_{E}$ are obtained and integrated with visual representations to provide precisely aligned inputs for subsequent global dependency modeling.

#### 2.3.3. Multimodal Temporal Transformer Representation Learning Module

The multimodal temporal Transformer representation learning module operates on the aligned multimodal features and performs deep temporal dependency modeling and cross-modal interaction learning within a unified semantic space.

**Figure 2:**
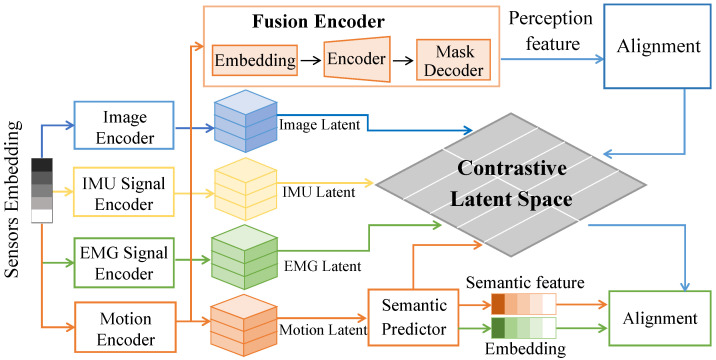
Schematic illustration of the multimodal temporal Transformer representation learning module, showing cross-modal interaction and long-range dependency modeling of aligned multimodal temporal features within a unified attention space.

As illustrated in Figure 2, the aligned modality representations are denoted as ${\hat{F}}_{S},\;{\hat{F}}_{I},\;{\hat{F}}_{E}\;\in \;R^{T\times C}$. These features are interleaved along the temporal dimension such that tokens from different modalities at the same time step become locally adjacent, forming a joint sequence $X^{\left(0\right)}\;\in \;R^{(3T)\times C}$. The backbone adopts a 4-layer Transformer encoder architecture to facilitate deep hierarchical feature abstraction. Each encoder layer is meticulously structured with a multi-head self-attention (MHSA) mechanism consisting of 8 parallel attention heads, a hidden dimensionality $d_{model}\;=\;256$, and a position-wise feed-forward network (FFN) with an inner-layer dimension of 1024.

Each layer consists of pre-layer normalization (Pre-LN), multi-head cross-modal self-attention, and FFN sublayers with residual connections:

(9) $$U^{\left(\ell \right)}\;=\;X^{\left(\ell -1\right)}\;+\;A^{\left(\ell \right)}\left(\mathrm{LN}(X^{\left(\ell -1\right)})\right),$$

(10) $$X^{\left(\ell \right)}\;=\;U^{\left(\ell \right)}\;+\;F^{\left(\ell \right)}\left(\mathrm{LN}(U^{\left(\ell \right)})\right).$$

To explicitly capture both intra-modal temporal evolution and inter-modal coupling, modality relation bias and relative temporal bias are incorporated into attention weight computation:

(11) $$P\;=\;\mathrm{RowSoftmax}\left(QK^{⊤}\;+\;B_{time}\;+\;B_{modal}\right),$$

(12) Z = PV.

To prevent over-smoothing and enhance training stability, the GELU activation function and a dropout rate of 0.1 are applied within each FFN block. Because attention weights constitute a probability distribution across rows, each output vector is a convex combination of input value vectors, satisfying

(13) $${∥\sum_{j}p_{j}v_{j}∥}_{2}\;\leq \;\sum_{j}p_{j}∥v_{j}{∥}_{2}\;\leq \;\max_{j}{∥v_{j}∥}_{2},$$

which ensures bounded aggregation and prevents uncontrolled noise amplification. Through stacked layers, any two temporal steps or modality tokens become connected via attention pathways, establishing a global receptive field in the representation space. The final sequence $X^{\left(L\right)}$ is reorganized by temporal aggregation to produce $H\;\in \;R^{T\times C}$, encoding posture structure variation, kinematic rhythm, and muscle activation dynamics.

#### 2.3.4. Uncertainty-Aware Fusion Module

The uncertainty-aware fusion module operates after multimodal temporal Transformer representation learning and performs reliability estimation and adaptive weighted aggregation of high-level modality-specific representations $H_{S},\;H_{I},\;H_{E}\;\in \;R^{T\times C}$. The module follows a sequential structure comprising feature recalibration, uncertainty estimation, dynamic weight generation, and weighted fusion.

**Figure 3:**
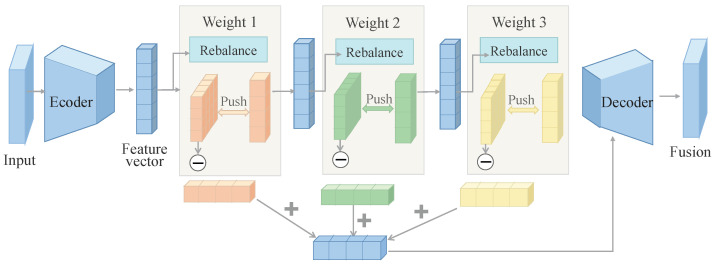
Schematic illustration of the uncertainty-aware fusion module, demonstrating adaptive confidence-based weighting and integration of multimodal features to enhance overall robustness.

As illustrated in Figure 3, each modality feature is first processed by a modality-specific uncertainty encoder consisting of two time-distributed perceptron layers with nonlinear activation. The temporal energy distribution of modality features is formulated as

(14) $$u_{t}^{\left(m\right)}\;=\;\phi \left(h_{t}^{\left(m\right)}W_{1}\right)W_{2},$$

and the corresponding uncertainty measure is defined as

(15) $$\sigma_{t}^{\left(m\right)}\;=\;\log\left(1\;+\;\exp(u_{t}^{\left(m\right)})\right).$$

Fusion weights are generated through normalized inverse confidence:

(16) $$\omega_{t}^{\left(m\right)}\;=\;\frac{\exp\left(-\sigma_{t}^{\left(m\right)}\right)}{\sum_{j}\exp\left(-\sigma_{t}^{\left(j\right)}\right)},$$

and the fused representation is computed as

(17) $$z_{t}\;=\;\sum_{m}\omega_{t}^{\left(m\right)}h_{t}^{\left(m\right)}.$$

Assuming each modality provides an unbiased estimate of a latent representation $z_{t}^{*}$, the expected squared error becomes

(18) $$E\left[∥z_{t}\;-\;z_{t}^{*}{∥}^{2}\right]\;=\;\sum_{m}\omega_{t}^{(m)2}\sigma_{t}^{\left(m\right)},$$

which is minimized when weights are proportional to inverse uncertainty, consistent with the proposed formulation. A temporal smoothness regularization term

(19) $$L_{smooth}\;=\;\sum_{t}{∥z_{t}-z_{t-1}∥}^{2},$$

is further introduced to maintain temporal consistency. Through adaptive confidence-based collaboration, modality contributions are dynamically adjusted according to scene conditions, ensuring robust and semantically consistent representations for downstream motion state prediction tasks.

### 2.4. Experimental Setup

The experimental hardware platform was established based on a high-performance workstation equipped with an NVIDIA RTX 4090 GPU (24 GB VRAM), an Intel Xeon multi-core CPU, and 128 GB DDR4 memory. The software environment was built on Ubuntu 22.04 using Python 3.10 and the PyTorch 2.x deep learning framework. Data preprocessing was performed using NumPy 2.3.0 and SciPy 1.14.0 for signal filtering, and OpenCV 4.13 for video frame parsing. Specifically, all IMU and EMG signals underwent Z-score normalization and were processed through a Butterworth band-pass filter to eliminate motion artifacts, while skeletal coordinates were normalized relative to the root joint to ensure spatial consistency.

To provide a thorough and precise description of the model output, the proposed framework employs a multi-task prediction layer. For action recognition, the output is a probability distribution ${\hat{y}}_{cls}\;\in \;R^{C}$ generated via a Softmax function, where C = 12 represents the action categories. For motion phase prediction, the model outputs a sequence of frame-level phase labels ${\hat{y}}_{phase}\;\in \;\left\{P_{1},\;\ldots ,\;P_{n}\right\}$, enabling precise segmentation of movement stages. For posture stability evaluation, the model generates a continuous scalar score s ∈ [0, 1] through a Sigmoid activation, where a higher value indicates superior balance and rhythmic consistency. These outputs are jointly optimized to provide a comprehensive characterization of the user’s motion state.

In response to the requirement to clearly define the data used for training and evaluation, we implemented a strict subject-independent partition protocol. The dataset, comprising 9216 multi-view samples, was divided based on participant identity to ensure that the model is evaluated on entirely unseen subjects. Specifically, the training set consists of data from 70% of the participants, used for model weight optimization via backpropagation. The validation set comprises data from 10% of the participants, employed exclusively for hyperparameter tuning and early stopping to prevent overfitting. The test set (or evaluation set) consists of the remaining 20% of the participants, which is held out and used only for the final performance assessment. This clear separation ensures that no temporal samples from a participant in the training set appear during the evaluation phase, thereby providing a rigorous measure of the system’s generalization capability to new individuals.

To ensure the technical precision of the proposed model, the detailed architectural specifications are summarized in Table 4. For the training iterations, a five-fold cross-validation strategy was adopted on the training and validation subjects, and the final results are reported as the average across the five runs. Upon acceptance of the manuscript, the complete source code will be made publicly available at https://github.com/Aurelius-04/UADMF.git (accessed on 6 April 2026).

### 2.5. Baseline Models and Evaluation Metrics

In the selection of baseline models, representative approaches are comprehensively covered across three mainstream technical paradigms, including unimodal modeling, conventional multimodal fusion, and deep multimodal temporal learning, thereby establishing a systematic comparative framework. Among them, ST-GCN [42] relies on the topological structure of the human skeleton to model spatial dependencies among joints, enabling effective characterization of motion structural variations. CTR-GCN [43] further improves this by implementing a channel-wise topology refinement mechanism, allowing the model to learn dynamic joint correlations tailored to different feature channels for enhanced spatial discrimination. BiLSTM [44] performs dynamic modeling of IMU temporal signals through a bidirectional gated recurrent mechanism, capturing contextual information from both past and future time steps. Limu-BERT [45] introduces a Transformer-based representation learning approach for inertial signals, leveraging self-attention to capture deep temporal dependencies and robust features from IMU data. TCN [46] employs dilated convolutional structures to model multi-scale temporal patterns in EMG signals, facilitating efficient extraction of muscle activation rhythms while enhancing long-sequence modeling efficiency. Early Fusion (Concat + MLP) [47] realizes joint representation of multi-source information through feature-level concatenation, providing a unified input space for cross-modal collaboration. Late Fusion (Weighted Average) [48] integrates multimodal prediction outputs at the decision level via weighted aggregation, offering strong structural flexibility. Multimodal Transformer [49] leverages global self-attention mechanisms to model cross-modal interaction relationships and long-range temporal dependencies within a unified semantic space, delivering powerful joint representation capability for complex motion state perception.

Accuracy was adopted to measure overall classification correctness, F1-score was used to evaluate the balance between precision and recall, and mAP was employed to assess overall ranking capability in multi-class detection or phase recognition tasks. In addition, a stability metric [50] was introduced to characterize temporal smoothness and fluctuation of predictions along the time dimension, enabling comprehensive evaluation of multimodal temporal model performance. The mathematical formulations of the evaluation metrics are defined as follows:

(20) $$\mathrm{Accuracy}\;=\;\frac{TP\;+\;TN}{TP\;+\;TN\;+\;FP\;+\;FN},$$

(21) $$\mathrm{Precision}\;=\;\frac{TP}{TP\;+\;FP},\;\mathrm{Recall}\;=\;\frac{TP}{TP\;+\;FN},$$

(22) $$F1\;=\;\frac{2\cdot \mathrm{Precision}\cdot \mathrm{Recall}}{\mathrm{Precision}\;+\;\mathrm{Recall}},$$

(23) $$AP\;=\;\int_{0}^{1}P\left(r\right)\;dr,\;mAP\;=\;\frac{1}{C}\sum_{c=1}^{C}AP_{c},$$

(24) $$\mathrm{Stability}\;=\;1\;-\;\frac{1}{T-1}\sum_{t=2}^{T}\left|{\hat{y}}_{t}\;-\;{\hat{y}}_{t-1}\right|.$$

Here, TP, TN, FP, and FN denote true positives, true negatives, false positives, and false negatives, respectively. P(r) represents precision at recall level *r*. *C* denotes the number of classes, and $AP_{c}$ represents the average precision of class *c*. *T* denotes the temporal length, ${\hat{y}}_{t}$ represents the predicted output at time step *t*, and |·| denotes the vector norm.

## 3. Results and Discussion

### 3.1. Baseline Comparison and Computational Efficiency Analysis

The purpose of this set of experiments is to systematically validate the performance, robustness, and cost-efficiency advantages of the proposed framework. To ensure a fair and comprehensive assessment, all baseline models and the proposed framework were trained and evaluated on a unified training database consisting of 9216 multimodal temporal sequences, providing a rigorous performance-to-cost evaluation.

As shown in Table 5, while the proposed UADMF framework involves a larger parameter count (16.2 M) and higher inference latency (38.5 ms) due to its triple-modality encoders and alignment module, it achieves a superior balance between complexity and robustness. Specifically, the framework yields a significant gain in temporal Stability (0.958) and F1@25 (91.22%) compared to high-performance unimodal models like CTR-GCN. The average inference time of 38.5 ms remains well within the real-time processing threshold (approximately 50 ms), ensuring its feasibility for clinical deployment. Furthermore, the training process on the 9216-sample database demonstrates that the framework effectively leverages multimodal redundancy to suppress noise and jitter without requiring an excessively large database for convergence. In summary, the results confirm that the proposed framework offers a high-performance, robust, and cost-effective solution for motion state perception in complex biomedical environments.

### 3.2. Robustness Evaluation

The purpose of this experiment is to systematically evaluate the robustness of the proposed multimodal temporal motion perception framework under complex interference and information deficiency conditions. To ensure the reproducibility of these evaluations, we have strictly quantified the interference parameters. For “Visual Occlusion,” we simulated partial perception by randomly masking 30% to 50% of the skeletal joints in each frame. “Viewpoint Variation” was implemented by introducing random spatial rotations of $\pm 15^{\circ }$ to the skeletal coordinates to simulate perspective shifts. “Sensor Noise” was modeled by injecting additive white Gaussian noise (AWGN) into the raw IMU and EMG streams, maintaining a signal-to-noise ratio (SNR) of 20 dB. “Modality Absence” refers to a complete signal loss (100% dropout) for the specified modality during the entire inference sequence. Unlike the primary experiment that focuses on recognition accuracy under ideal conditions, this evaluation verifies the system’s reliability in practical deployment.

**Figure 4:**
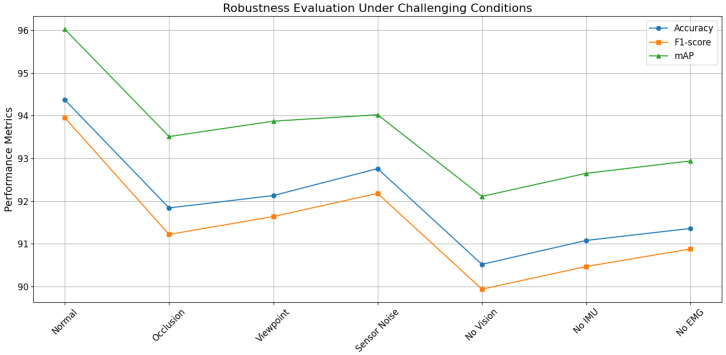
Robustness performance trends of the proposed model under occlusion, noise, viewpoint variation, and modality-missing conditions.

As shown in Table 6 and Figure 4, the model achieves peak performance under normal conditions. When various perturbations are introduced, all metrics decrease to varying degrees, yet the overall degradation remains controlled. The quantitative definition of these perturbations allows for a more rigorous assessment: even with a 50% joint occlusion rate or a significant 20 dB noise level, the accuracy remains above 91%, which underscores the effective error-suppression capability of our uncertainty-aware fusion module. The most pronounced decline occurs under visual occlusion, reflecting the importance of skeletal structural information. Performance degradation under viewpoint variation is comparatively limited, indicating stable structural modeling. The smallest decline appears under sensor noise, suggesting that the fusion stage exhibits strong tolerance to signal quality fluctuations. Modality-missing experiments further reveal differences in multimodal contributions. The largest performance drop occurs when the visual modality is removed, indicating its irreplaceable role in spatial structural constraints. From a mechanistic perspective, the robustness observed under these quantified noise and occlusion levels arises from the redundancy in multimodal attention. When a modality is corrupted, its contribution is automatically attenuated, while other modalities compensate through attention-based information propagation, thereby maintaining stable decision boundaries. This dual mechanism of cross-modal redundancy and adaptive confidence modulation enables consistently high performance, confirming the environmental adaptability of the proposed framework.

### 3.3. Ablation Study

The ablation experiments are designed to systematically validate the independent contribution and cooperative effect of key components in the proposed framework, with a specific focus on the reliability and robustness of the temporal alignment mechanism. By progressively removing or replacing core modules, performance variations are analyzed to demonstrate the architectural rationality. The variants include: (1) the full model; (2) w/o Cross-Modal Temporal Alignment Attention, which replaces the alignment module with standard linear interpolation; (3) w/o Temporal Alignment, which directly concatenates heterogeneous features without any synchronization or offset compensation; (4) w/o Multimodal Temporal Transformer; (5) w/o Uncertainty-Aware Fusion; and (6) w/o Modality Dropout.

As shown in Table 7, the full model achieves optimal performance across all metrics. The gain in accuracy is primarily attributed to the Cross-Modal Temporal Alignment Attention module. Comparing the full model to the w/o Cross-Modal Alignment variant, we observe a 1.56% drop in accuracy. This highlights that simply upsampling to a unified 1000 Hz grid using spline interpolation is insufficient to bridge the gap between 30 Hz skeletal sequences and 1000 Hz EMG signals. The learnable attention mechanism is the specific element that enables the model to resolve the sub-millisecond synchronization errors and neuromuscular latency, ensuring that kinematic variations are precisely correlated with muscle activation spikes. The system’s robustness is significantly enhanced by the Uncertainty-Aware Fusion module. When this module is removed, the accuracy decreases from 94.37% to 91.93%, and the Stability score drops to 0.926. This module is critical because it dynamically estimates modality reliability and adaptively allocates weights. In practical scenarios involving visual occlusion or sensor noise, this module suppresses unreliable streams and enhances stable ones, thereby preventing error propagation that would otherwise degrade the decision boundaries. Furthermore, the Multimodal Temporal Transformer backbone serves as the foundation for temporal smoothness; by capturing long-range dependencies and intra-modal interactions, it ensures a high Stability score of 0.958, effectively mitigating prediction flickering that occurs in non-aligned or non-Transformer-based architectures. In summary, the synergistic integration of these architectural elements provides a physiologically grounded invariant for robust motion perception under complex, asynchronous acquisition conditions.

### 3.4. Generalizability Validation on Public Dataset

To further verify the generalization capability of the proposed framework and ensure the architecture is not overfitted to the self-collected dataset, we conducted extensive evaluations on the UTD-MHAD public benchmark. UTD-MHAD is a widely recognized multimodal dataset comprising synchronized skeletal sequences and inertial measurement unit (IMU) signals. By adopting this dataset, we aim to demonstrate that the core components of our framework—specifically the modality-specific encoders, the cross-modal temporal alignment, and the uncertainty-aware fusion—maintain their superior performance across different hardware configurations and acquisition environments. For this experiment, all baseline models mentioned in our comparative study were re-implemented and evaluated under a subject-independent protocol to ensure a fair and rigorous comparison.

As shown in Table 8, the proposed framework consistently outperforms both unimodal and multimodal baselines. Among unimodal methods, CTR-GCN and Limu-BERT show significant improvements over traditional ST-GCN and BiLSTM, confirming the efficacy of dynamic topology refinement and Transformer-based temporal modeling. However, their performance remains lower than that of the fusion-based methods, highlighting the necessity of cross-modal collaboration. While Early and Late Fusion strategies provide baseline improvements, they are unable to effectively model the complex temporal offsets present in heterogeneous data. The Multimodal Transformer achieves higher accuracy by capturing long-range dependencies, yet it still lags behind our complete framework. The full proposed model achieves the highest accuracy of 92.15% and a stability score of 0.932, proving that the integration of temporal alignment and uncertainty-aware weighting effectively mitigates the performance degradation caused by cross-domain distribution shifts. These results confirm that our framework provides a robust and scalable solution for human motion monitoring that is applicable beyond the specific conditions of the original training environment.

### 3.5. Discussion

The proposed AI-driven multimodal framework represents a significant advancement over extant motion monitoring paradigms by establishing a unified semantic space for vision-based skeleton sequences, inertial rhythms, and muscle activation intensity. Compared to state-of-the-art methods such as Husformer and MTFT [34,35], which primarily focus on high-level feature fusion, our UADMF framework explicitly addresses the underlying synchronization issues inherent in heterogeneous sensing. Our results demonstrate a clear performance advantage over the strongest unimodal baseline, CTR-GCN [43], with a gain of approximately 5% in accuracy (94.37% vs. 89.45%) and a substantial improvement in temporal stability (0.958 vs. 0.898). This accuracy gain is critical for biomedical applications; for example, the 5% improvement translates to a significantly higher sensitivity in detecting neuromuscular compensatory patterns, where identifying the exact millisecond-level discrepancy between muscle firing (EMG) and limb displacement (Skeleton/IMU) is essential for clinical diagnosis.

The gains in accuracy and robustness are directly attributable to specific architectural innovations. The cross-modal temporal alignment attention module resolves the semantic displacement caused by the massive frequency discrepancy between 30 Hz video and 1000 Hz EMG signals. Furthermore, the uncertainty-aware fusion module provides the necessary error-suppression to maintain performance above 91% even when subjected to 50% joint occlusion or 20 dB sensor noise, a level of resilience not observed in traditional early or late fusion techniques. In terms of computational cost, the framework achieves a balance between complexity and real-time feasibility. While the model utilizes 16.2 M parameters—more than unimodal TCN or BiLSTM architectures—it maintains an inference latency of 38.5 ms, which is well within the 50 ms threshold required for real-time biomedical monitoring. During the training phase, the model demonstrated efficient convergence on the 9216-sample database. On a high-performance workstation equipped with an NVIDIA RTX 4090 GPU, the framework reaches convergence within 150 epochs, requiring approximately 5 h of total training time. This indicates that the architecture effectively leverages multimodal redundancy to learn generalized features without requiring excessively large datasets.

Regarding the relevance and transferability of our findings, the dataset utilized in this study—comprising fundamental movements such as walking, squatting, and lunging—represents a comprehensive set of biomechanical primitives that are common to a wide range of physical activities. By modeling the intricate relationships between skeletal kinematics, joint angular velocities, and neuromuscular activation during these representative tasks, the framework captures generalized motion features rather than task-specific patterns. This indicates a high degree of transferability to more complex athletic or clinical activities, such as stair climbing or sports-specific strength training, which share the same underlying flexion-extension and multi-joint coordination principles. Furthermore, the inclusion of diverse observational conditions, such as viewpoint variations and partial occlusions within our database, ensures that the learned multimodal representations are robust to environmental shifts. The framework’s ability to maintain high stability across these fundamental primitives suggests that the structure-rhythm-force coupling acts as a physiologically grounded invariant, providing a reliable foundation for monitoring unobserved physical activities in real-world biomedical applications.

### 3.6. Limitation and Future Work

Although stable recognition performance is achieved in complex motion scenarios, several aspects require further investigation. A primary limitation of this study lies in the scale and demographic diversity of the dataset, which is currently restricted to 32 healthy adult volunteers within a narrow age range of 22 to 35 years. While this cohort provides a baseline for technical validation, the findings may potentially over-claim clinical or biomedical applicability, as the results may not generalize to elderly populations or patients with significant musculoskeletal impairments. Furthermore, the dataset was primarily collected in controlled indoor environments; despite incorporating occlusion, noise, and viewpoint perturbations to enhance diversity, distribution discrepancies remain compared with large-scale outdoor sports, clinical rehabilitation wards, or crowded interactive environments. Generalization capability under broader open-world conditions requires further cross-scenario validation. Additionally, multimodal synchronization relies on unified acquisition systems; in practical deployment, clock drift and communication latency among heterogeneous devices may introduce more complex temporal misalignment, demanding higher real-time adaptability from alignment mechanisms. Furthermore, long-term wearing comfort and stability of IMU and EMG sensors may influence continuous monitoring quality, indicating the need for optimization in sensor layout and lightweight system integration. Future research may focus on multi-center collaborative data collection, weakly supervised or self-supervised temporal representation learning, edge-side real-time inference acceleration, and low-power wearable system integration to enhance deployability and long-term operational reliability in real-world complex environments.

## 4. Conclusions

This study targets the challenge that human motion states are difficult to be stably perceived under complex motion scenarios, where multi-source sensing data are characterized by temporal asynchrony, semantic misalignment, and quality fluctuations that hinder collaborative modeling. To address these issues, a unified multimodal temporal perception framework is constructed. By introducing modality-specific encoding mechanisms, deep feature representations of skeletal structures, inertial motion dynamics, and muscle activation signals are effectively extracted. On this basis, a cross-modal temporal alignment attention module is designed to explicitly alleviate temporal offsets induced by heterogeneous sampling mechanisms and physiological response delays. Furthermore, a multimodal temporal Transformer representation learning network is developed to model long-range motion dependencies and cross-modal interaction relationships within a unified attention space. In addition, an uncertainty-aware fusion mechanism is incorporated to dynamically allocate modality weights according to modality confidence, thereby suppressing noise interference while reinforcing reliable information contributions. Through this multi-level architectural design, systematic collaboration is achieved across temporal consistency modeling, semantic co-representation, and fusion reliability regulation. Comprehensive experimental results demonstrate that the proposed method achieves significant performance advantages on the multimodal human motion state perception dataset, with overall Accuracy reaching 94.37%, F1-score reaching 93.95%, and mAP reaching 96.02%, while attaining an optimal Stability score of 0.958. Comparative experiments verify that the complete framework consistently outperforms single-modality models, conventional fusion strategies, and non-aligned multimodal Transformer architectures. Robustness evaluations further indicate that only limited performance degradation is observed under visual occlusion, viewpoint variation, sensor noise, and modality-missing conditions. Ablation studies additionally confirm that the cross-modal temporal alignment module, the temporal Transformer backbone, and the uncertainty-aware fusion mechanism each contribute critically to overall performance improvement. Overall, high-accuracy, high-stability, and strongly generalized unified modeling is achieved under complex motion environments, providing reliable technical support for human motion state perception in practical scenarios such as rehabilitation assessment, athletic training, and intelligent human–machine interaction. Furthermore, large-scale deployment of the framework in rehabilitation and sports training may reduce labor-intensive assessment costs, improve resource allocation efficiency, and provide data-driven economic value for the digital transformation of the sports health industry. 

## Figures and Tables

**Table 1:** Summary of representative heterogeneous data fusion methods and their handling of recording frequencies.

Method/Paradigm	Fused Modalities	Fusion Technique	Frequency Heterogeneity Handling
Classical Estimation	IMU + Vision	Kalman Filter/EKF	Linear interpolation or state prediction
Recurrent Modeling	IMU + EMG	BiLSTM/GRU	Zero-padding or fixed-window segmentation
Graph-based Fusion	Skeleton + IMU	Adaptive GCN	Feature-level concatenation after resampling
Attention-based	Vision + Sensor	Standard Transformer	Pre-processing via spline interpolation
Proposed UADMF	Vision + IMU + EMG	Temporal Alignment Attention	Dynamic learnable offset modeling and asynchronous matching

**Table 2:** Summary of the multimodal human motion dataset collected in this study.

Modality	Sensor/Source	Sampling Rate	Data Volume
RGB Video	Multi-view Cameras	30 fps	9216 video clips
Skeleton Sequences	Pose Estimation (derived)	30 fps	9216 sequences
IMU Signals	Wrist/Ankle/Waist IMUs	100 Hz	27,648 signal streams
EMG Signals	Surface EMG (multi-channel)	1000 Hz	18,432 signal streams
Annotated Motion Labels	Manual + Semi-auto	–	12 action classes

**Table 3:** Demographic characteristics of the participants (N = 32).

Parameter	Mean ± SD/Range	Unit
Age	28.5 ± 4.2/22–35	years
Sex (Male/Female)	16/16	-
Height	171.2 ± 7.4/155–186	cm
Weight	66.4 ± 10.8/48–92	kg

**Table 4:** Architectural specifications and hyperparameter configurations of the proposed framework.

Module	Parameter Name	Value/Configuration
Skeleton Encoder	Architecture	Temporal Transformer
	Layer/Attention Heads	4 Layers/8 Heads
	Hidden/FFN Dimension	256/1024
IMU Encoder	Architecture	Bidirectional LSTM
	Hidden Layers/Size	2 Layers/128 units
EMG Encoder	Architecture	Temporal Convolutional Network (TCN)
	Dilation Factors	[1, 2, 4, 8]
Alignment & Fusion	Cross-attention Heads	4 Heads
	Modality Dropout (*p*)	0.2
	Fusion Mechanism	Uncertainty-aware Weighting
Training Protocol	Optimizer/Weight Decay	AdamW/$1\times 10^{-2}$
	Learning Rate Schedule	Cosine Annealing ($1\times 10^{-4}\to 1\times 10^{-6}$)
	Batch Size/Epochs	32/150
	Dropout Rate	0.1

**Table 5:** Comprehensive comparison with baseline models (Mean ± SD). Performance is evaluated across five-fold cross-validation.

Method	Accuracy (↑)	F1-Score (↑)	F1@25 (↑)	mAP (↑)	Stability (↑)	Params (M)	Time (ms)
ST-GCN (Skeleton)	86.21 ± 0.85	85.74 ± 0.91	82.53 ± 1.12	88.03 ± 0.74	0.872 ± 0.015	3.1	12.4 ± 0.8
CTR-GCN (Skeleton)	89.45 ± 0.62	88.92 ± 0.75	85.81 ± 0.94	91.15 ± 0.58	0.898 ± 0.012	1.46	18.2 ± 1.1
BiLSTM (IMU)	83.95 ± 1.15	83.41 ± 1.28	80.14 ± 1.42	85.62 ± 1.05	0.854 ± 0.021	2.5	10.1 ± 0.5
Limu-BERT (IMU)	87.32 ± 0.78	86.84 ± 0.84	83.76 ± 1.02	89.06 ± 0.65	0.881 ± 0.018	12.1	24.5 ± 1.4
TCN (EMG)	81.37 ± 1.45	80.92 ± 1.56	77.45 ± 1.78	83.18 ± 1.32	0.841 ± 0.025	0.85	8.7 ± 0.4
Early Fusion	88.64 ± 0.95	88.12 ± 1.02	85.02 ± 1.15	90.37 ± 0.82	0.891 ± 0.014	5.2	22.1 ± 1.5
Late Fusion	89.08 ± 0.88	88.65 ± 0.94	85.64 ± 1.08	90.94 ± 0.76	0.897 ± 0.013	5.1	23.4 ± 1.6
Multi-Transformer	91.73 ± 0.54	91.26 ± 0.62	88.47 ± 0.85	93.18 ± 0.48	0.921 ± 0.009	14.8	32.6 ± 2.1
**Proposed (Full)**	94.37 ± 0.42	93.95 ± 0.51	91.22 ± 0.68	96.02 ± 0.35	0.958 ± 0.006	**16.2**	38.5 ± 1.8

**Table 6:** Robustness evaluation of the proposed model under challenging conditions (Mean ± SD across five-fold cross-validation).

Setting	Accuracy (↑)	F1-Score (↑)	mAP (↑)	Stability (↑)
Normal condition	94.37 ± 0.42	93.95 ± 0.51	96.02 ± 0.35	0.958 ± 0.006
Visual occlusion (30–50% joints)	91.84 ± 0.95	91.22 ± 1.04	93.51 ± 0.82	0.936 ± 0.012
Viewpoint variation ($\pm 15^{\circ }$ shift)	92.13 ± 0.78	91.64 ± 0.85	93.87 ± 0.64	0.941 ± 0.010
Sensor noise (SNR = 20 dB)	92.76 ± 0.65	92.18 ± 0.72	94.02 ± 0.55	0.948 ± 0.008
Missing Vision modality (100% loss)	90.52 ± 1.12	89.94 ± 1.25	92.11 ± 1.05	0.918 ± 0.018
Missing IMU modality (100% loss)	91.08 ± 1.05	90.47 ± 1.18	92.65 ± 0.92	0.924 ± 0.015
Missing EMG modality (100% loss)	91.36 ± 0.98	90.88 ± 1.02	92.94 ± 0.88	0.927 ± 0.014

**Table 7:** Ablation study of key components in the proposed framework (Mean ± SD across five-fold cross-validation), validating the necessity and reliability of temporal alignment.

Variant	Accuracy (↑)	F1-Score (↑)	F1@25 (↑)	mAP (↑)	Stability (↑)
Full model	**94.37** ±0.42	93.95±0.51	91.22±0.68	96.02±0.35	0.958±0.006
w/o Cross-Modal Alignment Attention	92.81 ± 0.72	92.24 ± 0.85	88.45 ± 1.05	94.37 ± 0.62	0.934 ± 0.012
w/o Temporal Alignment (Direct)	89.42 ± 1.25	88.96 ± 1.38	84.17 ± 1.65	91.28 ± 1.15	0.892 ± 0.024
w/o Multimodal Transformer	90.76 ± 0.98	90.11 ± 1.12	86.52 ± 1.35	92.54 ± 0.88	0.912 ± 0.018
w/o Uncertainty-Aware Fusion	91.93 ± 0.82	91.28 ± 0.94	87.94 ± 1.18	93.62 ± 0.75	0.926 ± 0.015
w/o Modality Dropout	93.12 ± 0.65	92.54 ± 0.78	89.87 ± 0.95	94.85 ± 0.55	0.941 ± 0.011

**Table 8:** Generalizability performance comparison on the UTD-MHAD public dataset (Mean ± SD across five-fold cross-validation).

Method	Accuracy (↑)	F1-Score (↑)	mAP (↑)	Stability (↑)
ST-GCN (Skeleton only)	84.32 ± 1.05	83.85 ± 1.14	86.14 ± 0.92	0.856 ± 0.018
CTR-GCN (Skeleton only)	87.56 ± 0.82	87.12 ± 0.95	89.37 ± 0.74	0.882 ± 0.014
BiLSTM (IMU only)	81.04 ± 1.35	80.56 ± 1.48	82.85 ± 1.22	0.831 ± 0.025
Limu-BERT (IMU only)	85.12 ± 0.94	84.67 ± 1.02	86.94 ± 0.81	0.865 ± 0.019
Early Fusion (Concat + MLP)	86.47 ± 1.12	85.93 ± 1.24	88.26 ± 0.98	0.874 ± 0.016
Late Fusion (Weighted Average)	86.92 ± 1.02	86.41 ± 1.15	88.73 ± 0.88	0.879 ± 0.015
Multimodal Transformer	89.38 ± 0.75	88.92 ± 0.88	91.24 ± 0.62	0.903 ± 0.011
**Proposed (Full Model)**	92.15±0.58	91.73±0.64	93.86±0.45	0.932±0.008

## Data Availability

The original contributions presented in this study are included in the article. Further inquiries can be directed to the corresponding author.
